# The Immunologic Profile of Vitamin D and Its Role in Different Immune-Mediated Diseases: An Expert Opinion

**DOI:** 10.3390/nu14030473

**Published:** 2022-01-21

**Authors:** Sandro Giannini, Andrea Giusti, Salvatore Minisola, Nicola Napoli, Giovanni Passeri, Maurizio Rossini, Luigi Sinigaglia

**Affiliations:** 1 Clinica Medica 1, Department of Medicine, University of Padova, 35128 Padova, Italy; 2 Metabolic Bone Disease Unit & Fracture Liaison Service, Department of Medical Specialties, Regional Health Trust 3, 16125 Genova, Italy; andreagiusti6613@gmail.com; 3 Department of Clinical, Internal, Anaesthesiology, and Cardiovascular Sciences, Sapienza University of Rome, 00185 Rome, Italy; salvatore.minisola@uniroma1.it; 4 Division of Endocrinology and Diabetes, Universita Campus Bio-Medico di Roma, 00128 Rome, Italy; n.napoli@unicampus.it; 5 Unit of Clinica e Terapia Medica, Department of Medicine and Surgery, University of Parma, 43126 Parma, Italy; giovanni.passeri@unipr.it; 6 Rheumatology Unit, Department of Medicine, University of Verona, 37134 Verona, Italy; maurizio.rossini@univr.it; 7 Division of Rheumatology, ASST Gaetano Pini-CTO, 20122 Milano, Italy; reumatologia@luigisinigaglia.it

**Keywords:** vitamin D, immune-mediated disease, cholecalciferol, COVID-19

## Abstract

Historically, vitamin D is recognized as an essential component for the maintenance of the musculoskeletal system. The immunomodulatory role of vitamin D in health and disease has gained much interest in recent years due to the many pathologies that share underlying immunological features where vitamin D has been shown to exert a potential role. Evidence from pre-clinical studies show that vitamin D elicits biological effects on both the innate and adaptive immune systems. Furthermore, in vivo studies have shown that administration of vitamin D can lead to changes in or the development of a range of immune-related diseases. This encourages the hypothesis that data derived from clinical and epidemiological studies connect vitamin D with the incidence and severity of many immune-mediated disorders such as rheumatoid arthritis, diabetes, and infectious diseases. Since some other immune-mediated diseases share similar features to that of viral infection such as COVID-19, in this review, we examined these other areas and the role of vitamin D in these diseases.

## 1. Introduction

Vitamin D is essential for the homeostatic regulation of calcium [1], and reduced vitamin D intake can result in vitamin D deficiency or inadequate levels, impacting bone metabolism and leading to an increase in the secretion of parathyroid hormone (PTH) and subsequent increase in bone resorption [2,3]. Vitamin D is synthesized (or introduced into the body with food), undergoes a first hydroxylation in the liver, and is transformed into 25-hydroxyvitamin D3 [25(OH)D_3_] or calcidiol. Despite the fact that 25(OH)D is not the active form of vitamin D, it is measured in the clinical setting to establish vitamin D status due to its long half-life (2–3 weeks) [4,5,6]. Calcidiol is further hydroxylated to its active form, calcitriol; 1,25-dihydroxyvitamin D [1,25(OH)_2_D_3_], this second hydroxylation, mainly occurs in the kidney [7]. The activation process is regulated by the circulation of serum levels of PTH, calcium, and phosphorus. In turn, 1,25(OH)2D_3_ controls PTH secretion through a negative feedback mechanism.

Vitamin D deficiency is a very common condition worldwide, particularly in elderly and osteoporotic subjects, in which the risk of bone fragility increases [2,8,9,10]. In addition to playing an essential role in maintaining bone health, vitamin D is also recognized for its immunomodulatory effects [11,12,13,14]. Both the vitamin D receptor (VDR) and metabolizing enzymes, such as 1-α-hydroxylase, are expressed by different types of immune cells including macrophages, T cells, dendritic cells, monocytes, and B cells [15,16,17,18,19], and evidence from pre-clinical studies has shown that vitamin D exerts biological effects on both the innate and adaptive immune systems [11,13,14]. Extra-renal 1-α-hydroxylase is not upregulated by PTH; therefore, 1,25(OH)_2_D_3_ production is dependent upon levels of the substrate 25(OH)D_3_ and may be regulated by inflammatory signals, such as lipopolysaccharide (LPS) and cytokines [20,21]. In vivo studies have shown that the administration of vitamin D can lead to changes in or the development of a range of immune-related diseases [11,12,13,22]. This favors the idea that clinical and epidemiological data link vitamin D with the incidence and severity of many immune-mediated disorders such as rheumatoid arthritis [23], diabetes [24], and infectious diseases [25,26]. A summary of immune-mediated diseases commonly associated with vitamin D deficiency is presented in Table 1.

To date, evidence documenting the protective effects of vitamin D supplementation is derived from many observational studies and meta-analyses of clinical trials for the prevention of viral acute respiratory tract infection (ARTI) [27,28,29]. Low vitamin D status (i.e., <20 ng/mL [7], measured as plasma levels of vitamin D, 25(OH)D) is prevalent worldwide and frequently observed in regions of Northern latitudes, but also in Southern countries [9].

Indeed, the benefit of vitamin D supplementation in patients with COVID-19 only remains speculative and is partially supported by limited data from observational studies [30,31,32] and three clinical trials [33,34,35]. 

The aim of the present narrative review was to provide an update on available evidence in some specific autoimmune/inflammatory diseases that share common immunological features with each other in order to further our understanding of the role of vitamin D in these settings. Each of the authors contributing to this review has different specialist expertise in different immune-mediated diseases as well as extensive experience in the use of vitamin D. The role of vitamin D in the immune system and with auto-immune diseases such as infectious diseases (including COVID-19), rheumatic diseases, and diabetes mellitus is discussed. 

## 2. Vitamin D and the Immune System

The first indirect evidence of a potential role of vitamin D and its metabolites in immune regulation probably lies in an ancient paper published more than 150 years ago. The beneficial effects of pure, fresh cod liver oil in the treatment of 234 cases of tuberculosis were described [36]. The scientific community had to wait over 100 years to enter the era of the link between vitamin D and the immune system with the discovery of 1,25-dihydroxy vitamin D (1,25(OH)_2_D) specific high-affinity receptors in human leukocytes [37] and in human peripheral blood mononuclear cells [38]. The subsequent step was the demonstration that the expression of 1-α hydroxylase, the final enzyme for the activation of the terminal metabolite of the vitamin D biologic system, is not restricted to the kidney proximal tubule cells but is upregulated by immunologic stimuli also in the monocytic-macrophage lineage [38]. Since these historical discoveries, a large body of evidence has accumulated on the mechanistic role for vitamin D in the regulation of the immune response (either innate or adaptive) through its active metabolite, calcitriol [1,25(OH)_2_D_3_] [14,39,40]. A summary of the effects of vitamin D on innate and adaptive immunity is presented in Table 2. Two main observations support this hypothesis: The vitamin D receptor (VDR) is expressed in almost all immune cells, including B and T lymphocytes, monocytes, macrophages, and dendritic cells, and some immune cells are able to convert 25-hydroxy-vitamin D [25(OH)D] to 1,25(OH)_2_D [14,39,40].

### 2.1. Innate Immunity

The discovery of a physiological role of vitamin D in the regulation of the innate immune response began with the finding that activated macrophages and dendritic cells express CYP27B1 (the gene encoding for 1-α hydroxylase) and the VDR gene [21,48]. In 2006, a pivotal study demonstrated that toll-like receptor (TLR) activation of human monocytes and macrophages with a synthetic *M. Tuberculosis*-derived lipopeptide led to a shift in the gene expression profile of these cells enhancing the expression of VDR and CYP27B1, with an increase in the endogenous production of 1,25(OH)_2_D from circulating 25(OH)D [20]. In the same study, 25(OH)D_3_ (calcifediol) supplementation on TLR-stimulated human monocytes in culture strongly enhanced the expression of cathelicidin, a natural antibacterial peptide, thus supporting the idea that 1,25(OH)_2_D through binding to VDR could regulate the innate immune response. These observations, together with an IL-15-dependent, CYP27B1-induced stimulation of macrophage development and differentiation and the well-known excessive 1,25(OH)_2_D_3_ production by macrophages in several granulomatous diseases, led to the assumption that vitamin D can act as a potent stimulator of mechanisms associated with pathogen elimination. Furthermore, 1,25(OH)_2_D in monocytes and hepatocytes inhibit the expression of hepcidin, another natural antibacterial protein directed to suppress the transmembrane protein, ferroportin, thus inhibiting iron export from cells and limiting iron supply to microorganisms to sustain their growth [49].

Dendritic antigen-presenting cells express VDR and are modulated by 1,25(OH)_2_D towards a more tolerogenic phenotype through a delay in their maturation, thus limiting the ability to present antigen to T cells. In mature dendritic cells, VDR expression is reduced [50] with a decrease in sensitivity to 1,25(OH)_2_D, allowing an initial presentation of antigen to T cells. The inhibition of cell maturation acts as a barrier to the overstimulation of T cells. Neutrophils express VDR but lack CYP27B1 [51], indicating that these cells may preferentially function as a hormonal target for 1,25(OH)_2_D. Existing data indicate that vitamin D can induce the formation of neutrophil extracellular traps (NETs) and acts on primary human neutrophils as a modulator of the inflammatory response by lowering inflammatory neutrophil-derived cytokine production [52]. Collectively, these data indicate that vitamin D reinforces bacterial killing by these cells, while restricting a neutrophil-induced inflammatory response [53]. Furthermore, 1,25(OH)_2_D is also able to influence eosinophil function through the downregulation of interleukin (IL)-15, the pivotal cytokine involved in the recruitment of these cells [54], and lowers immunoglobulin-E (IgE)-dependent mast cell activation [55]. Natural killer (NK) cells play an intermediate role between innate and adaptive immunity [56]. Several studies confirm that vitamin D also exerts a regulatory role on this family of T lymphocytes by modulating their cytotoxicity, cytokine secretion, and degranulation [57].

Other evidence suggests that 1,25(OH)_2_D may directly modulate the expression of several cytokines implicated in innate immunity. In co-cultures of infected macrophages, vitamin D induces the expression of IL-1-beta and IL-8 [58] and, in infected human peripheral blood mononuclear cells, downregulates the expression of other proinflammatory cytokines such as IL-6, tumor necrosis factor-(TNF)-α, and interferon (IFN)-γ [59]. These data confirm an important role of 1,25(OH)_2_D in enhancing a prompt response to infection together with a modulating effect on the acute inflammatory response.

### 2.2. Adaptive Immunity

Besides the indirect consequences on T-cell differentiation and function derived from the effects of vitamin D on innate immune cells, 1,25(OH)_2_D acts as a regulator of mature T cells by altering the balance between T helper (Th)1 and Th2 cell differentiation. Specifically, 1,25(OH)_2_D enhances the expression of IL-4 and strongly inhibits IFN-γ production from naïve CD4+ T cells in culture, thus showing the capacity to inhibit Th1 and reciprocally promote Th2 cell differentiation [44]. This effect may suggest a possible preventive or therapeutic role of 1,25(OH)_2_D in several Th1 cell-driven diseases, such as multiple sclerosis, type 1 diabetes mellitus (T1D), inflammatory bowel diseases, and rheumatoid arthritis (RA). Another study performed in a mouse model of human Crohn disease confirmed that calcitriol with or without dexamethasone upregulates Th2 markers and is able to reduce not only Th1 differentiation but also the Th17-driven response, indicating that vitamin D may exert some of its effects on inflammation and autoimmunity through the regulation of IL-17 expression in T cells [45]. A reduction of Th17 cells is usually counterbalanced by an increase in T-regulatory cells’ (T-regs) reciprocal axis. The 1,25(OH)_2_D_3_ together with transforming growth factor beta (TGF-β) is able to induce forkhead box P3 expression in naïve CD4+ T cells, promoting T-regs’ differentiation [46], and to increase the production of the anti-inflammatory cytokine IL-10 from CD4+/CD25+ T-regs [60] with potential beneficial effects in several autoimmune diseases. With the aim to better understand the molecular mechanisms involved in the orderly shutdown and retraction of CD4+ type Th1 cell response, Chauss et al. recently analyzed the bronchoalveolar lavage fluid CD4+ T cells of patients with COVID-19 and identified an autocrine/paracrine vitamin D loop that allows Th1 cells to activate and respond to vitamin D as part of a complex shutdown program repressing IFN-γ and enhancing IL-10 [61]. Indeed, the molecular pathways identified in this study may yield important information to aid in the development of novel therapeutic approaches to accelerate the shutdown program of hyper-inflammatory cells in COVID-19 patients.

Human active B-lymphocytes carry VDR as well as 1-α-hydroxylase, suggesting that vitamin D may strongly also influence this family of immune cells [47]. B-cell response is influenced by vitamin D via an inhibition of the ongoing proliferation of activated B-cells, induction of B-cell apoptosis, and inhibition of the generation of plasma cells. As a net result of these effects, vitamin D acts as an inhibitor of immunoglobulin secretion, also suggesting a possible role of vitamin D in B-cells-related disorders.

Experimental data accumulated in the past three decades provide convincing evidence for an immunomodulatory effect of vitamin D either on innate or on adaptive immunity [14,62]. To mention a paradigmatic example, in RA, vitamin D exerts effects against the intrinsic disease mechanisms, which include Th1 polarization, lower expression of Th2 cytokines, higher immunoglobulin production, enhanced Th17 differentiation, and lower T-regs’ activation and function. Based on these anti-inflammatory properties of vitamin D, a protective effect on several inflammatory and autoimmune diseases has been hypothesized by numerous reports demonstrating (mainly retrospective studies) low circulating levels of 25(OH)D in a high proportion of patients with chronic inflammatory conditions. In this respect, it is important to address two critical points. First of all, a mechanism of reverse causality induced by chronic diseases cannot be ruled out. Secondly, data derived from several recent studies support the hypothesis that systemic inflammation lowers 25(OH)D concentrations, thus contributing to the low circulating levels observed in patients suffering from chronic inflammatory diseases. In this context, in a recent experimental study based on a human endotoxemia model reproducing systemic inflammation in vivo, circulating levels of 25(OH)D significantly decreased shortly after initiation of a bolus of *E. coli-*derived lipopolysaccharide [63].

## 3. Vitamin D and Infectious Diseases

As we have previously alluded to, 1,25(OH)_2_D exerts several direct and indirect effects on innate immunity (see Section 2) by directly affecting antimicrobial activity and by influencing gut microbiota composition. Compelling evidence suggests that 1,25(OH)_2_D is capable of enhancing the production of defensin β2 and cathelicidin antimicrobial peptide (CAMP) by macrophages/monocytes; of up-regulating CAMP in keratinocytes, epithelial, intestinal, lung and corneal cells, and placenta trophoblasts; of increasing the chemotaxis, autophagy, and phagolysosomal fusion of innate immune cells; and of enhancing corneal, intestinal, and epithelial barrier function (e.g., maintaining tight and gap junctions) [19,64]. Moreover, macrophages formed after IL-15 stimulation have been shown to respond to vitamin D by increasing their antimicrobial activity (in contrast to those obtained after IL-10 stimulation that are weakly influenced by vitamin D) [19].

The effects of vitamin D on the adaptive immunity are relevant mainly, but not only, to autoimmune and rheumatic diseases [14,19,40,65]. As we have already mentioned, 1,25(OH)_2_D has been shown to down-regulate the immune response mediated by Th1 cells, to up-regulate Th2 cells’ activity, to suppress Th 17 formation and activity, to increase Tregs’ function, to modulate differentiation and antibody production of B cells, and to induce apoptosis and cell cycle arrest of proliferating B cells [14,19,40,65].

Historically, as we have alluded to previously in this review, the antimicrobial properties of vitamin D have been recognized for their protective effects on *M. Tuberculosis* disease. The 25(OH)D levels have been shown to influence the incidence of tuberculosis disease and its clinical course as well [66,67,68]. More recently, the potential beneficial effects of vitamin D in several other infectious diseases have been observed, including hospital-acquired *Clostridium Difficile* infection (CDI), influenza, ARTIs, COVID-19 infection, and sepsis (including mortality due to sepsis) [29,64,69,70,71,72].

Upala et al. investigated the association between vitamin D deficiency and sepsis in hospitalized patients in a systematic review and meta-analysis of observational studies [70]. The authors observed a higher risk of sepsis in patients with serum levels of 25(OH)D <20 ng/mL measured before or during hospitalization compared to those without vitamin D deficiency. Another meta-analysis also revealed an independent association between severe vitamin D deficiency (below 10 ng/mL) and increased mortality in adult patients with sepsis [71].

Several studies investigated the potential relationship between CDI and vitamin D. Particularly interesting are the results of a meta-analysis of epidemiological studies examining the association between serum 25(OH)D concentrations and CDI severity or recurrence. The study demonstrated a higher odds of severe CDI in subjects with lower 25(OH)D compared to those with higher 25(OH)D [72]. On the other hand, there was no significant association between 25(OH)D status and CDI recurrence.

Both epidemiological studies and RCTs demonstrated that, by raising 25(OH)D concentrations in winter, the risk of developing influenza can be reduced, particularly in school children and infants [64]. A recent review of the literature, including five RCTs, concluded that the beneficial effect of vitamin D supplementation on the risk of developing influenza was seen for doses of cholecalciferol of around 1200 IU per day [64]. According to these results, an observational study examining the relationship between serum 25(OH)D concentration and incidence of ARTIs (mainly influenza) demonstrated a protective effect of vitamin D for concentrations above 38 ng/mL [73].

The most compelling data on the antimicrobial effects of vitamin D are those related to ARTIs [29]. In this area, the results of a systemic review and meta-analysis of individual patients’ data, investigating the effects of vitamin D supplementation on the risk of ARTI, are particularly relevant. Vitamin D deficiency was demonstrated to significantly reduce the incidence of ARTI (outcome: proportion of participants with a least one ARTI and rate of ARTI). In subgroup analysis of patients receiving daily or weekly vitamin D, the protective effect was stronger in those subjects with baseline serum 25(OH)D concentrations below 10 ng/mL. Interestingly, the beneficial effects of vitamin D were seen in subjects receiving daily or weekly vitamin D without additional bolus doses, but not in those receiving one or more bolus doses. All these findings were, in general, also confirmed by considering potential confounders [29]. Recently, these findings were confirmed in another meta-analysis that showed that the protective effect of vitamin D was associated with daily doses of 400–1000 IU given for up to 12 months [74].

Nitric oxide (NO) functions as a pro-inflammatory mediator and plays a recognized role in a range of inflammatory [75] diseases including respiratory diseases [76]. The association between NO and vitamin D is also recognized. Vitamin D has been shown to act as a transcription factor for the regulation of endothelial NOS in mice [77] and improve NO-dependent vasodilation in adipose tissue arterioles from bariatric surgery patients [78]. Vitamin D has also been shown to increase the expression of inducible nitric oxide synthase (iNOS) in patients with COVID-19, a relationship discussed further in Section 6 [79].

Overall, the results of basic and clinical studies strongly support the role of vitamin D in preventing and reducing the severity, and possibly the complications, of several infectious diseases. In general, and considering potential confounding variables, data derived from these studies point towards beneficial effects in subjects with varying degrees of vitamin D deficiency at baseline who receive appropriate doses/dosing regimens of vitamin D.

## 4. Vitamin D in Rheumatic Auto-Immune Diseases

Epidemiological studies have unequivocally confirmed a high prevalence of hypovitaminosis D in several auto-immune rheumatic diseases (AIRDs). On average, patients with RA, psoriatic arthritis (PsA), ankylosing spondylitis (AS), systemic sclerosis (SS), and lupus (SLE) appear to have 25(OH)D values lower by at least 8–10 ng/mL compared to those of healthy control groups [80,81,82,83,84,85]. In the CARMA study [80], the vitamin D status of 2234 patients with RA, PsA, and AS was compared with that of 667 healthy subjects. Vitamin D deficiency (defined as 25(OH)D < 20 ng/mL) fluctuated between 40 and 41% in patients with RA, PsA, and AS compared to 27% in healthy subjects (*p* < 0.001).

In patients affected by RA, the association between RA and hypovitaminosis D also emerged as particularly strong by multivariate analysis (OR = 1.5, 95% CI: 1.1–2.0) [80]. The high incidence of hypovitaminosis D in patients with RA was clearly confirmed by a meta-analysis performed on 15 observational studies (1100 RA patients and 1000 healthy controls) [83]. The authors observed considerably lower mean 25(OH)D values in RA patients compared to those in the control group and further found that deficiency was significantly higher in patients with RA (55% in RAs vs. 33% in healthy subjects; OR = 2.5, 95% CI: 1.1–5.3).

Similar studies on patients with SLE or SS produced the same results [81,82,84,85]. Islam et al. conducted a review of several studies on the prevalence of hypovitaminosis D in patients with SLE [84]. The average 25(OH)D value in SLE patients was generally about 10 ng/mL lower compared to the control group. Analogous results were found in another meta-analysis conducted on data in SS and hypovitaminosis D (six studies, 554 SS patients, and 321 healthy subjects) [85]. The standardized average difference between patients with SS and healthy subjects was about 9 ng/mL, with some variability linked to the characteristics of the SS. Lower vitamin D levels have been described in patients with antiphospholipid syndrome (APS) compared to healthy controls, particularly in patients with thrombotic disease [86].

Even though data described so far clearly indicate a relationship between hypovitaminosis D and some AIRDs, they are unable to define a cause–effect relationship. In other words, these data do not clarify the possible pathogenetic link between prolonged 25(OH)D deficiency and disease onset. In the case of vitamin D, it is difficult to establish a cause–effect relationship: To do so would require long-term longitudinal studies performed on the general population. In this context, then, data regarding AIRDs’ incidence in healthy subjects as a function of either the baseline levels of 25(OH)D or of exposure to UVB or of cholecalciferol intake are clearly insufficient. Two large prospective cohort studies have described the correlation between exposure to UVB and the risk of developing RA: the Nurses’ Health Study (NHS) and the Nurses’ Health Study II (NHSII) [87].

The NHS, conducted on a population of more than 100,000 women, showed a lower incidence of RA in subjects who had a higher cumulative average exposure to UVB compared to women who had a lower exposure (HR = 0.8, 95% CI: 0.7–0.9) [87]. These results were not confirmed in the NHSII since UV-B was not associated with RA risk among younger women (RR = 1.12, 95% CI: 0.87–1.44). Differences in sun-protective behaviors (e.g., greater use of sun block in younger generations) may explain these differences. It should be noted that post hoc analysis of these two studies failed to confirm the relationship between circulating 25(OH)D serum levels or dietary intake of vitamin D and RA or SLE risk [88,89,90]. However, the Iowa Women’s Health Study, which investigated the incidence of RA as a function of vitamin D intake in a population of more than 29,000 women, showed that higher vitamin D intake (both via diet and supplementation) was associated with a reduced risk of RA (RR = 0.7, 95% CI: 0.4–1.0) [91]. Therefore, ad hoc studies need to be designed and carried out to further investigate the cause–effect relationship between hypovitaminosis D and AIRDs’ occurrence.

The existence of a relationship between vitamin D status [serum 25(OH)D] and disease activity or severity has been reported in several studies that were primarily (but not exclusively) carried out on patients with RA, SLE, and SS [23,81,82,83,92,93,94]. A summary of some recent studies evaluating the effect of vitamin D supplementation in patients with immune-mediated rheumatic diseases is available as supplemental material (Supplementary Table S1). Most studies that examined the relationship between 25(OH)D and disease activity in patients with RA showed an inverse correlation between vitamin D status and disease activity of 28 joints (DAS28), visual analogue scale (VAS), and/or erythrocyte sedimentation rate (ESR) [23,83,92,93]. In the COMORA study, for instance, involving 1413 RA patients, average DAS28 values in subjects with normal vitamin D levels were considerably lower compared to subjects with hypovitaminosis D [93]. A similar relationship (inverse correlation) was also found for antibodies to citrullinated protein antigens (ACPAs) by Wang et al. [92]. Vitamin D deficiency has been associated with more active and severe disease and may predict disability and radiographic progression in early RA patients [95]. Additionally, for SS and SLE patients, clinical data showed an inverse correlation between 25(OH)D and disease activity or clinical outcomes (scleroderma ulcers) [81,82,94]. Regarding SS patients, for example, Caimmi et al. [82] analyzed the relationship between the variation of 25(OH)D values over time and the incidence of digital ulcers in 65 SS patients. They found that a 25(OH)D reduction (in 48% of patients) during a 5-year follow-up was associated with higher risk for developing digital ulcers (OR = 16.6, 95% CI: 1.7–164.5) [82]. Another study, which investigated average 25(OH)D values as a function of disease activity measured by the Systemic Lupus Erythematosus Disease Activity Index (SLEDAI) in 199 SLE patients, found a progressive decrease of 25(OH)D in tandem with a progressive worsening of the SLEDAI [81]. In the ASAS-COMOSPA international study, vitamin D deficiency was observed in 51.2% of the patients with SpA, and vitamin D deficiency was independently associated with the presence of radiographic sacroiliitis (OR = 2.1, 95% CI: 1.3–3.3) [96].

Overall, the epidemiological and clinical findings described here have opened the way toward the hypothesis that a reduction in disease activity and perhaps even improved clinical results can be attained by using cholecalciferol supplementation in AIRDs patients with vitamin D deficiency [97]. Promising data were obtained from exploratory studies that used cholecalciferol supplementation in RA patients. Recent prospective studies that administered cholecalciferol 60,000–100,000 IU per month in RA patients showed beneficial effects on VAS and/or DAS28 [98,99]. The most important point is the demonstrated different effects of cholecalciferol on DAS28 and VAS depending on baseline 25(OH)D levels: The most beneficial effects of cholecalciferol on DAS28 were found in patients with baseline 25(OH)D > 20 ng/mL, while its greatest effects on VAS were in patients with baseline 25(OH)D < 20 ng/mL [99]. However, in a recent study analyzing 1180 RA patient records, vitamin D supplementation did not provide additional benefit for anti-rheumatic treatment [100]. These data support the need for prospective, randomized, controlled trials (RCTs) to evaluate the role of vitamin D supplementation in treating RA. In a double-blind, placebo-controlled RCT in patients with RA and vitamin D deficiency, high doses of cholecalciferol resulted in a statistically significant improvement in functional disability after 6 months, even if clinically not relevant [101]. The conclusions of two recent systematic reviews were that the correction of hypovitaminosis D may have a beneficial effect on pain perception, on disease control, or on incidence of recurrence, but that the current evidence is still not strong enough to consider vitamin D supplementation as a disease-modifying anti-rheumatic drug [102,103].

On the relationship between vitamin D supplementation and SLE disease activity, there are currently two RCTs available that found that vitamin D supplementation was able to reduce disease SLE activity [104,105], while another RCT failed to observe any significant result [106]. The weakness of the available data is probably due to several limitations: the number of patients, uncertain dosage, duration of the follow-up, and baseline 25(OH)D serum values [97].

To date, vitamin D supplementation trials for AIRDs have varied substantially in terms of sample size, characteristics of patients, disease duration and severity, concomitant medication regimen, type of/duration of supplementation regimen, number of outcomes, and period over which these outcomes were measured. A recent meta-analysis identified nine RCTs evaluating the use of vitamin D supplementation for ≥3 months in rheumatic diseases, including five studies in RA patients [102]. In RA, there was a decrease in the rate of disease flare, VAS, and DAS28 with vitamin D supplementation; however, all outcome measures failed to reach statistical significance.

In general, within the limits dictated by the complexity and heterogeneity of AIRDs, data from the literature appear to unambiguously confirm a role of vitamin D in diseases such as RA, SS, and SLE. Its effect with regard to other AIRDs (PsA, AS, and SpA) seems less clear, mainly because of the scarcity of published studies and their modest quality. In general, we can state that serum 25(OH)D levels seem to influence the activity and severity of some AIRDs and can possibly also have an effect on certain clinical outcomes; less clear is the cause–effect relationship in AIRDs’ pathogenesis.

## 5. Vitamin D and Diabetes Mellitus

### 5.1. Type 1 Diabetes

T1D is an autoimmune disease determining beta cell loss and lifelong insulin dependence [107]. Age of onset usually occurs at a very young age, implying a high risk of developing diabetes-related complications, including bone fragility [108]. In the last three decades, data derived from a range of studies have shown a possible interplay between vitamin D deficiency and T1D.

VDR and 1α-hydroxylase are expressed in the beta cell, implying a direct effect of vitamin D on pancreatic homeostasis [109]. Several lines of evidence have shown a direct effect of vitamin D on pancreatic calcium inflow/outflow and insulin gene transcription [109]. Moreover, the influence of vitamin D in T1D relies on the autoimmune pathogenesis of this condition. It is well known, in fact, that vitamin D also exerts immunomodulatory effects [24,110,111], preventing or suppressing the autoimmune process [112,113]. Experimental models have demonstrated that vitamin D at high doses prevents insulitis and onset of experimental diabetes by acting on the defective suppressor cellular function or cytokine expression modulation [24,110,111]. Convincing clinical evidence was provided by a Finnish study about 20 years ago, showing a reduced incidence of T1D in children who were supplemented since birth with vitamin D [114]. Vitamin D supplementation was associated with a decreased frequency of T1D vs. no supplementation (RR = 0.12, 95% CI: 0.03–0.51), suggesting that adequate vitamin D supplementation in newborns may reverse the increasing trend in the incidence of T1D [114]. Other subsequent studies have documented that new-onset T1D patients have reduced levels of 1,25(OH)_2_D and 25(OH)D compared with healthy controls [115].

However, a RCT, using 0.25 µg of calcitriol per day or placebo for 1 year, proved no effect on insulin secretion, insulin sensitivity, or insulin requirement compared with placebo [116]. A further sub-analysis of the same cohort showed that 0.25 µg of calcitriol did not improve markers of bone turnover [117]. Similarly, Walter et al. found no effect of calcitriol supplementation in newly diagnosed T1D in insulin requirement and C-peptide at 9 and 18 months [118]. These negative data may be explained either by the low dose of calcitriol or by the lack of efficacy of vitamin D once the disease has developed. Therefore, vitamin D may prevent the onset of the disease, at least in selected populations, but without changing the fate of the disease if administrated after the diagnosis. In support of this hypothesis, Norris et al. showed a lower risk of developing islet autoimmunity in infants with higher vitamin D levels, with a 43% reduced risk in infants with vitamin D > 50 nmol vs. those with <50 nmol [119]. According to the same study, a crucial role is played by the VDR genotype, with lower islet autoimmunity risk based upon number of minor alleles of the VDR rs7975232 SNP. These data were, in part, confirmed in another cohort showing that increased 25(OH)D levels at birth decrease T1D risk, depending on VDR genotype [120]. Taken together, these data may indicate that specific genetic traits lower VDR expression and, by consequence, inhibit T-cell proliferation and increase the risk of autoimmunity.

### 5.2. Type 2 Diabetes

Although T2D is not an immune-mediated disease, vitamin D may also modulate inflammation associated with insulin resistance, leading to a reduction in the transport of glucose stimulated by insulin itself [121,122]. Effects of vitamin D supplementation and insulin resistance in the clinical setting are contradictory [123,124,125,126,127,128,129,130]. In a small hyperinsulinemic euglycemic clamp study, supplementation with cholecalciferol (11,200 IU/day for 2 weeks followed by 5600 IU for an additional 10 weeks) or placebo for 12 weeks [131], showed no significant changes in insulin sensitivity, inflammation, blood pressure, lipid balance, or glycated hemoglobin (HbA1c). Many observational studies have investigated the association between low vitamin D and the higher risk of T2D [132,133,134,135], and likely a positive effect of vitamin D on lowering the risk of diabetes may be exerted only in subjects who are vitamin D deficient. A preventive role in the onset of T2D attributed to vitamin D was supported by Barbarawi et al. in a recent study, showing that vitamin D supplementation in patients with prediabetes treated with ≥1000 IU/day of vitamin D significantly reduced the risk of diabetes’ incidence compared to the placebo group (RR: 0.88, CI: 0.79–0.99) [136].

In conclusion, although several lines of evidence have shown a positive, direct effect of vitamin D on beta cell regulation, only a few and inconclusive clinical studies have explored a possible effect on preventing both T1D and T2D. Polymorphisms of VDR gene may exert an important role, but genetic profiling of at-risk subjects is not clinically suitable. Diabetic patients are often vitamin D deficient and adequate supplementation should be always advised. However, according to recent consensus statements, there is insufficient evidence to recommend specific indications and how often vitamin D should be measured in diabetic patients. In general, diabetic patients need higher doses of oral vitamin D supplementation to achieve desired 25(OH)D levels. Therefore, a daily dose of at least 800 IU of vitamin D (up to 4000 IU per day) should be recommended [137,138]. While a possible positive effect on disease progression cannot be proven yet, vitamin D supplementation is crucial for bone health and bone fragility prevention, a common complication of diabetes.

## 6. Potential Role of Vitamin D in the COVID-19 Pandemic

In December 2019, some hospitals in Wuhan, China, reported many patients with atypical pneumonia of unknown cause. Broncho-alveolar lavage samples were collected from these patients and utilized to infect human airway epithelial cells. Owing to the 85% identity with bat-SL-CoVZC45 coronavirus and 80% identity to SARS Corona-virus, this new, isolated virus was named as novel coronavirus 2019 (2019-nCOV) later renamed as SARS-CoV-2; the related disease was named COVID-19 by the World Health Organization [139].

The first case of severe disease from SARS-CoV-2 (Severe Acute Respiratory Syndrome-CoronaVirus-2), later named COVID-19 (CoronaVirus Disease-19), was reported in the city of Wuhan, China, in January 2020 [140]. Subsequently, the viral infection and the disease spread rapidly to many geographical areas of the world and, in March 2020, the disease was recognized as a pandemic by the World Health Organization [141]. As of 14 December 2021, just under 270,031,622 confirmed cases have been recorded worldwide since the start of the pandemic, with 5,310,502 deaths [139].

Across many countries around the world, infection and disease have been characterized by successive waves, also in relation to the contagion containment measures currently adopted by various countries. Patients with COVID-19 typically present with symptoms and signs of severe infectious respiratory disease, increased leukocytes, and frequent lymphocytopenia and inflammation parameters [142]. Interstitial pneumonia of varying severity usually becomes evident. A large number of people infected with SARS-CoV-2 may, in fact, remain asymptomatic or develop very mild symptoms.

Unfortunately, a subset of infected individuals develops a disease so severe that it requires hospitalization. About 20% of these subjects present respiratory conditions that require transfer to the intensive care unit (ICU) [143]. In these subjects, mortality can be very high, particularly in those who belong to the most advanced age groups and have important comorbidities [144].

What Is The Relationship between Vitamin D and COVID-19?

To date, no real therapy has been identified for the treatment of SARS-CoV-2 infection and, although many vaccines are now available with proven efficacy [145], the scientific community is looking very carefully at any drug capable of slowing viral replication and/or improving the course of the disease [146].

It is clear that a drug that is 100% effective for the treatment of SARS-CoV-2 patients (independently of age and severity of the disease) is not available at present. In this context, we now analyze the potential use of vitamin D.

The activation of the signaling pathway of the VDR seems to generate positive effects in Acute Respiratory Distress Syndrome (ARDS) [147], inducing a mitigation of the so-called “cytokine storm”, thus playing an important immuno-modulatory and anti-inflammatory role [148].

The relationship between NO and vitamin D has also been observed in the context of COVID-19. Gönen et al. (2021) evaluated the effectiveness of vitamin D3 supplementation on outcome in COV€ID-19 patients [79]. They retrospectively analyzed 867 cases and showed that patients without comorbidities, without vitamin D treatment and 25(OH)D <30 ng/mL had a 1.9-fold increased risk of hospitalization of greater than 8 days compared to patients with comorbidities and vitamin D treatment. Vitamin D treatment decreased the mortality rate 2.14-fold. They also performed correlation analysis of specific serum biomarkers with 25(OH)D and revealed that iNOS, IL-1β, IFN-γ, cathelicidin-LL37, and intercellular adhesion molecule-1 may be involved in the regulation of the action of vitamin D in COVID-19. Moreover, iNOS levels were higher in all COVID-19 cases compared with controls. However, serum 25(OH)D and iNOS levels were not correlated in healthy controls but negatively correlated in patients not receiving vitamin D supplementation and positively correlated in patients who received vitamin D supplementation. These results corroborate two other studies that observed increased blood nitrate and nitrite levels (metabolites reflecting total NO production) in COVID-19 patients compared to healthy subjects [149] and higher serum nitrate levels in COVID-19 patients who died (*n* = 11) compared to those who survived (*n* = 42) [150].

The possible protective role of vitamin D supplementation is supported by numerous observational studies and by meta-analyses of clinical trials concerning the prevention of ARVIs [27]. An insufficient vitamin D status has been proposed as a risk factor for acute, virus-induced respiratory diseases [29,66]. A compromised vitamin D status is, however, common in many countries [151]. This has drawn attention to a possible relationship between hypovitaminosis D and infection with SARS-CoV-2 and COVID-19 [152,153]. Ilie et al. [154], analyzing the data of 20 European countries, observed a negative correlation (*r* = −0.44, *p* = 0.05) between serum vitamin D (56.8 ± 10.6 nmol/L) and the number of COVID-19 cases per million inhabitants.

In the same study, COVID-19 mortality was higher in subjects with low vitamin D levels. A dose-response pattern was seen in a cohort of >190,000 patients in whom SARS-CoV-2 infection had been correlated with serum vitamin D levels in the previous 12 months [155]. In this cohort, an inverse correlation was observed between vitamin D levels and positivity for SARS-CoV-2.

Furthermore, the virus positivity rate was significantly higher in 39,190 patients with vitamin D < 20 ng/mL (12.5%, 95% CI: 12.2–12.8%) compared to 27,870 patients with “adequate” serum values (30–34 ng/mL) (8.1%, 95% CI: 7.8–8.4%) and to subjects with serum levels > 55 ng/mL (5.9%, 95% CI: 5.5–6.4%). In a multivariate analysis, those who had a serum vitamin D < 20 ng/mL demonstrated a 54% higher positivity rate than subjects with normal values. The risk of contracting SARS-CoV-2 progressively decreased until reaching values of 55 ng/mL. Many other studies have further confirmed the relationship between hypovitaminosis D, SARS-CoV-2 infection, and COVID-19 mortality.

In recent months, two Italian studies have helped to strengthen the hypothesis of a relationship between hypovitaminosis D and COVID-19. A retrospective study of 137 patients, with a mean age of 65 years, hospitalized for COVID-19 showed a prevalence of hypovitaminosis D of 100%. However, those who died had significantly lower serum vitamin D values than those who survived the disease (12 ng/mL vs. 8 ng/mL, *p* < 0.01). In a multivariate logistic regression analysis, vitamin D levels correlated inversely with in-hospital mortality (OR, 0.91; 95% CI: 0.85–0.98; *p* < 0.01) [156].

In a retrospective study, conducted at the University of Verona, on a cohort of 61 patients (mean age 69 years), hospitalized because of COVID-19, 72.1% were vitamin D-deficient (<20 ng/mL) and 57.4% had serum 25(OH)D < 15 ng/mL. Patients with respiratory failure (PaO_2_ < 60 mmHg) demonstrated lower vitamin D values than subjects with normoxemia (PaO_2_ ≥ 60 mmHg) (13.3 ng/mL vs. 20.4 ng/mL, respectively, *p* = 0.03). Hypovitaminosis D was associated with a 3-fold greater risk of hypoxemia and an increase in CRP and the degree of dyspnea [157].

In a prospective study undertaken in 103 in-patients (mean age 66 years; 70% male) admitted to a hospital in Milan for severely symptomatic COVID-19, low 25(OH)D levels were negatively correlated with elevated levels of IL-6 and emerged as independent predictors of COVID-19 severity and mortality [158].

A really different aspect is whether there may be a link between the administration of vitamin D and the clinical course of COVID-19. In other words, whether cholecalciferol can have positive effects on the evolution of COVID-19. Indeed, in the past 2 years, there has been accumulating evidence from several observational studies and a few clinical trials in this context (Supplementary Table S2).

A RCT by Murai and colleagues [33] did not provide encouraging results. However, the cohort, although including a larger number of subjects (*n* = 240), had a rather young average age (about 56 years), and therapy with vitamin D (cholecalciferol; 200,000 IU) was administered over 10 days after the onset of symptoms. The length of hospitalization was the main outcome and was no different between subjects on active treatment and subjects on placebo. However, it was only 7 days long, indicating that the selected subjects did not have a particularly severe disease development. Among the secondary outcomes, mortality and need for ICU transfer did not differ between groups. However, once again, the mortality rate of these patients was low overall (around 6%), as was the need for transfer to ICU, around 18% of patients. In these studies, only a minor proportion of patients had baseline vitamin D levels < 20 ng/mL. Another double-blind RCT [34] gave much more encouraging results. Subjects infected with SARS-CoV-2 and with vitamin D deficiency (<20 ng/mL, mean value about 9 ng/mL) received over 400,000 IU in about 7 days. Patients treated with native vitamin D, compared to those on placebo, previously negative for the virus experienced a significant decrease in fibrinogen, one of the potential markers of disease severity.

A retrospective study conducted on elderly hospitalized patients (average age of 74 years) in the UK demonstrated how a high dose of cholecalciferol (> 200,000 IU) was able to decrease mortality in patients hospitalized for COVID-19 [159].

Vitamin D supplementation was also associated with significantly reduced mortality during the COVID-19 pandemic in a cohort of 157 residents of an Italian nursing home [160].

In a prospective study by Annweiler et al. [30], performed on very elderly (88 ± 5 years) and very frail subjects hospitalized for COVID-19, the effect of vitamin D supplementation was evaluated. They divided the 77 occupational patients into three groups: Group 1, COVID-19 patients admitted to the hospital, but who had received during the year previous cholecalciferol doses ranging from 50,000 IU per month or up to 100,000 IU every 2–3 months; Group 2, patients not on stable supplementation with native vitamin D, but who had received 80,000 IU of cholecalciferol after admission to hospital due to COVID-19; and Group 3, patients with the same clinical characteristics, but who had never received vitamin D, nor did they take it while in hospital. The primary outcome was mortality during hospitalization and the secondary outcome was from the Ordinal Scale for Clinical Improvement Score for COVID-19 in Acute Phase (OSCI). Given the high burden of morbidity and fragility of the patients, a long series of covariates were used to adjust for confounding in their analysis. In Group 1, 93% of patients survived to 14 days, compared with 81% in group 2 and 68 in group 3 (*p* < 0.05). Considering Group 3 (untreated) as a reference, the HR for mortality at 14 days, largely corrected for possible confounding factors, was equal to 0.07 (*p* < 0.05) for Group 1 (treated in the year before admission with cholecalciferol) and 0.37 (*p* > 0.05) for Group 2 (treated only during hospitalization). Furthermore, Group 1 was associated with a better OSCI than Group 3 (*p* < 0.05). The authors concluded a positive effect of cholecalciferol therapy, capable of inducing a less severe COVID-19 reaction and increasing survival in frail elderly.

In another quasi-experimental study from the same group [31], 82.5% of 57 nursing home residents who received bolus vitamin D3 supplementation either in the week following the suspicion or diagnosis of COVID-19 or during the previous month survived during a mean follow-up of 36 days compared with only four out of nine residents (44%) without this therapy. Despite the small number of cases, the association was highly statistically significant, with an adjusted hazard ratio of 0.11 (95% CI: 0.03–0.48).

Similar results were also seen from our retrospective study of 91 patients hospitalized for COVID-19, of advanced age (74 years), with a range of important comorbidities and very low basal levels of vitamin D (36 mmol/L, range interquartile 16–60) [32]. In 36 subjects (39.6%), cholecalciferol was administered at a dose of 400,000 IU orally, divided into 2 consecutive days at the time of admission. The remaining 55 subjects (60.4%) were not treated with vitamin D. The study aimed to assess whether the proportion of patients experiencing ICU transfer and/or death could be affected by vitamin D intake. During a follow-up period of approximately 14 days, 27 (29.7%) patients were transferred to the ICU and 22 (24.2%) died. Overall, 43 patients (47.3%) experienced “Death or ICU transfer”. The statistical analysis revealed that the “weight” of comorbidities (represented by the history of cardiovascular disease, chronic obstructive pulmonary disease, chronic renal failure, neoplastic disease not in remission, diabetes mellitus, hematological diseases, and endocrine diseases) significantly changed the protective effect of vitamin D on the study objective, so that the greater the number of comorbidities present, the more evidence of benefit induced by vitamin D was seen. In particular, the risk of undergoing “Death/Transfer to ICU” was reduced by about 80% compared to subjects who had not taken it (OR 0.18, IC: 0.04–0.83, *p* < 0.05, after correction for multiple confounding factors). In conclusion, in elderly, highly comorbid patients affected by COVID-19, cholecalciferol significantly reduced mortality and severity of the disease. This hypothesis-generating study warrants a formal evaluation (i.e., clinical trial) of the potential benefit that cholecalciferol can offer in these comorbid COVID-19 patients.

A recent systematic review was performed by Dramè et al. with the aim to evaluate current evidence on the relationship between vitamin D supplementation and elderly COVID-19 patients [161]. A total of 707 studies were initially considered in this analysis and 11 observational studies were included. In studies comparing patients with vitamin D deficiency to patients without vitamin D deficiency, those without vitamin D deficiency were found to have better primary clinical outcomes. This systematic review appears to support an association between vitamin D deficiency and the risk of COVID-19 in aged people. Furthermore, patients who are deficient in vitamin D appear to have greater risk of adverse outcomes. Thanks to its ease of administration combined with a rare occurrence of side effects, the inclusion of vitamin D as a preventive strategy for some specific viral diseases would appear to be an attractive option.

Brenner elegantly provided an update of recent epidemiological and intervention studies on a possible role of vitamin D supplementation for preventing severe COVID-19 cases and deaths. The authors concluded that “despite the inherent limitations and remaining uncertainties, accumulating evidence strongly supports widespread vitamin D supplementation, in particular of high-risk populations, as well as high-dose supplementation of those infected” [28].

Furthermore, achieving optimal vitamin D status could benefit COVID-19 patients in preventing falls and fractures during or after hospitalization [162]. It is recommended to give daily or weekly dosing regimens of 800 IU/day of cholecalciferol (or equivalents) [163,164] up to 4000 IU/day as needed (i.e., vitamin D deficient patients) [165].

Given the level of importance derived from evidence accumulated so far, several studies are ongoing, including controlled RCTs to confirm the importance of the use of vitamin D in patients with COVID-19. At present, at least three large RCTs [166,167,168] are in the advanced stage and at least two of these [166,167] offer very robust outcomes. It is, therefore, possible that in a reasonably short time a confirmation of the role of vitamin D (and cholecalciferol in particular) can be reached as a possible drug that can assist in the fight against the pandemic generated by SARS-CoV-2, which for a long time now has afflicted almost all the inhabitants of our planet, with consequences, even today, too often fatal.

## 7. Conclusions

In this narrative review, we discussed the available evidence from RCTs and real-life observational studies on the potential role of vitamin D in selected immune-mediated diseases. In different auto-immune diseases, such as rheumatic diseases and diabetes as well as infectious diseases (including COVID-19), we can observe a generalized reduction in circulating 25(OH)D levels in these patients compared to healthy individuals. The clinical meaning and the reasons explaining reduced levels in 25(OH)D levels in inflammatory illnesses are not completely understood but could be related to a drop in vitamin D binding protein or to a possible increase in 1-α hydroxylation of 25(OH)D [169]. Evidence from the molecular analysis of pathways involving vitamin D and the regulation of the immune response in COVID-19 patients also provide important information [61]. Generally, outcome measures are improved following vitamin D supplementation, particularly at higher and daily doses in patients already deficient in vitamin D.

It is also important to bear in mind that data derived from many clinical studies evaluating the association between vitamin D and various diseases need to be interpreted with caution, due to the presence of several confounding variables [170,171]. It should also be noted that many of the trials did not specifically recruit only vitamin D-deficient patients and that supplementation of “native” vitamin D may be different from giving calcitriol or 25(OH)D. Typical confounders may include age, study duration, high BMI, low physical activity, and baseline comorbidities. In addition, the association could be due to reverse causation in some circumstances [171] such as severe sepsis or systemic inflammation in COVID that can lead to leakage of albumin and vitamin D binding protein and consequently lead to low levels of 25(OH)D [172,173,174,175]. However, even though these considerations are pertinent, we believe that in-depth discussion were outside the scope of the present review.

The fascinating theme of immune regulation by vitamin D, based on a large body of experimental data, still needs validation on clinical grounds since the therapeutic use of vitamin D in supplementation trials has so far shown only modest benefit. In this respect, there is still a need for large RCTs in order to confirm whether vitamin D adequacy can reduce the incidence and severity of several infections and of different autoimmune diseases. Results from undergoing clinical trials (particularly in COVID-19 patients) are eagerly awaited.

## Figures and Tables

**Table 1:** Immune-mediated diseases commonly associated with vitamin D deficiency.

Infectious/Pulmonary Diseases	Skin Disease	Rheumatic Diseases	Metabolic	Other
Sepsis	Psoriasis	Rheumatoid arthritis	Type-1 diabetes	Multiple sclerosis
Tuberculosis	Urticaria	Psoriatic arthritis		
COPD	Dermatitis	Ankylosing spondylitis		
Asthma		Systemic sclerosis		
		Lupus		

COPD = chronic pulmonary obstructive disease.

**Table 2:** Effects of vitamin D on innate and adaptive immunity.

Function/Cells	Effect	Reference
Innate immunity		
Macrophage differentiation	Increased	Koeffler et al. 1984 [41]
Bacterial killing	Increased	Liu et al. 2006 [20]
Dendritic cells’ maturation	Decreased	Penna et al. 2000 [42]
Antigen presentation	Decreased	Griffin et al. 2000 [43]
Adaptive immunity		
Th1 cytokines	Decreased	Boonstra et al. 2001 [44]
Th2 cytokines	Increased	Boonstra et al. 2001 [44]
Th17 differentiation	Decreased	Daniel et al. 2008 [45]
T-regs’ differentiation	Increased	Chambers et al. 2014 [46]
B-cells	Decreased proliferation Induction of apoptosis Inhibition of plasma cell generation Inhibition of immunoglobulin secretion	Chen et al. 2007 [47]

## Data Availability

Not applicable.

## Supplementary Materials

The following are available online at https://www.mdpi.com/article/10.3390/nu14030473/s1, Table S1: Recent clinical studies evaluating the effect of vitamin D supplementation in patients with immune-mediated rheumatic diseases, Table S2: Recent clinical studies evaluating the effect of vitamin D supplementation in patients hospitalized for COVID-19.
