# Supplementary material for: The Immunologic Profile of Vitamin D and Its Role in Different Immune-Mediated Diseases: An Expert Opinion
Source: Nutrients. 2022 Jan 21;14(3):473. doi: 10.3390/nu14030473 (PMC8838062; doi:10.3390/nu14030473)
Supplement: Supplementary file 1 [file nutrients-14-00473-s001.zip › nutrients-1541218-supplementary.pdf]

Table S1. Recent clinical studies evaluating the effect of vitamin D supplementation in patients with immune-mediated rheumatic diseases.

Author, year	Study design, follow-up period	Patients, N	Treatment	Outcome	Result
Chandrashekara et al. 2017[1]	Open label, 3 months	Rheumatoid arthritis, N=73; 25(OH)D3 <20 ng/mL at baseline + DAS28-CRP >2.6	60,000 IU/week for 6 weeks then 60,000 IU/month for 3 months	Improvement in DAS28-CRP, vitamin D status	Decrease in DAS28-CRP and increase in vitamin D >20 ng/mL in the treatment group
Buondonno et al. 2017[2]	Double-blind randomised controlled trial, 3 months	Rheumatoid arthritis, N=39	Methotrexate + glucocorticoid vs. methotrexate + glucocorticoid + 300,000 IU (one dose)	Cell phenotypes, osteoclast precursors, inflammatory cytokines, clinical parameters at 3/12 months	Reduced IL-23 and increased global health score in vitamin D supplemented group
Guan et al. 2020 [3]	Meta-analysis of 6 randomized controlled trials, 12-24 weeks	Rheumatoid arthritis, N=438	8000–50,000 IU/week of oral vitamin D or equivalent or oral 1,25(OH)2D	Disease activity measures	Vitamin D supplementation resulted in a significant improvement in the DAS28 (weighted mean difference (WMD) = -0.41, 95% CI (-0.59, -0.23), P < 0.001), ESR (WMD = -3.40, 95% CI (-6.62, -0.18), P = 0.04) and TJC (WMD = -1.44, 95% CI (-2.74, -0.14), P = 0.03) but not in pain VAS.
Nguyen et al. 2020 [4]	A subgroup meta-analysis of 2 randomized controlled trial, 3 weeks to 1 year	Rheumatoid arthritis, N=252	Oral 50,000 IUs/week of vitamin D3 or 0.5 μg/day of alfacalcidol [1-hydroxyvitamin D3]	Risk of flare in patients in remission	Vitamin D supplementation resulted in an insignificant reduction in flares.
Zheng et al. 2019[5]	A meta-analysis of 5 randomized controlled trials, 3 to 12 months	Systemic Lupus Erythematosus, N=490	200–50,000 IUs/week of vitamin D3 or equivalent	Disease activity measures	Vitamin D3 supplementation resulted in a decrease in the fatigue severity scale scores in patients with SLE in a meta-analysis of 2 trials (n=79; SMD: -1.179, 95% CI: -1.90 to -0.46).

*in treated arm. CI = confidence interval; CRP =C-reactive protein; DAS28: disease activity score in 28 joints; ESR = erythrocyte sedimentation rate; HR = hazard ratio; ICU = intensive care unit; IU = international units; OR = odds ratio; RR = relative risk; TJC = tender joint count; WMD; weighted mean difference; WOMAC = Western Ontario and McMaster Universities Osteoarthritis Index

References

1. Chandrashekara, S.; Patted, A. Role of Vitamin D Supplementation in Improving Disease Activity in Rheumatoid Arthritis: An Exploratory Study. *Int J Rheum Dis* **2017**, *20*, 825–831, doi:10.1111/1756-185X.12770.
2. Buondonno, I.; Rovera, G.; Sassi, F.; Rigoni, M.M.; Lomater, C.; Parisi, S.; Pellerito, R.; Isaia, G.C.; D'Amelio, P. Vitamin D and Immunomodulation in Early Rheumatoid Arthritis: A Randomized Double-Blind Placebo-Controlled Study. *PLOS ONE* **2017**, *12*, e0178463, doi:10.1371/journal.pone.0178463.
3. Guan, Y.; Hao, Y.; Guan, Y.; Bu, H.; Wang, H. The Effect of Vitamin D Supplementation on Rheumatoid Arthritis Patients: A Systematic Review and Meta-Analysis. *Front Med (Lausanne)* **2020**, *7*, 596007, doi:10.3389/fmed.2020.596007.
4. Nguyen, Y.; Sigaux, J.; Letarouilly, J.-G.; Sanchez, P.; Czernichow, S.; Flipo, R.-M.; Soubrier, M.; Semerano, L.; Seror, R.; Sellam, J.; et al. Efficacy of Oral Vitamin Supplementation in Inflammatory Rheumatic Disorders: A Systematic Review and Meta-Analysis of Randomized Controlled Trials. *Nutrients* **2020**, *13*, E107, doi:10.3390/nu13010107.
5. Zheng, R.; Gonzalez, A.; Yue, J.; Wu, X.; Qiu, M.; Gui, L.; Zhu, S.; Huang, L. Efficacy and Safety of Vitamin D Supplementation in Patients With Systemic Lupus Erythematosus: A Meta-Analysis of Randomized Controlled Trials. *Am J Med Sci* **2019**, *358*, 104–114, doi:10.1016/j.amjms.2019.04.020.

Table S2. Recent clinical studies evaluating the effect of vitamin D supplementation in patients hospitalized for COVID-19.

Author, year	Study design, follow up length	Patients, N, age*	Treatment	Outcome	Result
Murai et al. 2020[1]	A multicentre, double-blind, randomized controlled trial	COVID-19, N=240 (16 treated), aged 50 years	200,000 IU "vitamin D3" for 7 days		Length of hospital stay, mortality rate, admission to ICU and mechanical ventilation requirement did not differ between groups. Vitamin D significantly increased 25(OH)D levels
Rastogi et al. 2020[2]	Randomised, placebo-controlled, 21 days	COVID-19, N=40 (16 treated), aged 50 years	80,000 IU cholecalciferol for 7 days	Proportion of patients with SARS CoV-2 RNA negative before day-21 and change in inflammatory markers	10/16 (62.5%) of treated patients achieved SARS-CoV-2 negativity vs 5/24 (20.8%) participants (p=0.018) in the control arm
Castillo et al. 2020[3]	Parallel pilot randomized open label, double-masked clinical trial, FU until admission to ICU, hospital discharge or death.	COVID-19, N=76 (50 treated), 53 years	Calcifediol, 0.53 mg and 0.266 mg at day 3 and 7	ICU, hospital discharge or death.	50 treated; 1 admitted to ICU vs 26 untreated and 13 admitted to ICU; univariate risk benefit: OR 0.02 (95% CI: 0.002- 0.17), multivariate: OR 0.03 (95% CI: 0.003-0.25)
Annweiler et al. 2020[4]	Prospective quasi-experimental, 36±17 days	COVID-19, N=66 (N=57 treated) aged 87.7 years	80,000 IU cholecalciferol	Mortality, and 9-point World Health Organization's ordinal scale	82.5 % in vitamin D group survived vs 44.4 % in comparator group (P=0.023). Mortality reduction: HR = 0.11 [95 % CI: 0.03-0.48], P=0.003. Vitamin D neg correlated with OSCI score for COVID-19 (β= -3.84 [95 % CI: -6.07; -1.62], P=0.001).
Annweiler et al. 2020[5]	Prospective quasi-experimental, 14 days	COVID-19, N=77 (N=16 in pz supp cholecalciferol after diagnosis) aged 85 years	80,000 IU cholecalciferol	14-day mortality, and 9-point World Health Organization's ordinal scale	No benefit from treatment after diagnosis but benefit from previous regular supplementation (N=29) (HR: 0.07, p = 0.017) vs. no treatment (N=32)
Tan et al. 2020[6]	Cohort observational study. All patients were followed through either to hospital discharge or day 30 from onset of symptoms, whichever was earlier	COVID-19, N=43 (17 treated), aged 58.4 years	Single daily oral 1,000-IU dose of vitamin D3 (cholecalciferol), 150 mg of magnesium oxide, and 500 µg vitamin B12 for ≤14 days.	Oxygen therapy, ICU over	DMB exposure was associated with odds ratios of 0.13 (95% CI, 0.03–0.59) and 0.20 (95% CI, 0.04–0.93) for oxygen therapy, intensive care support, or both
Ohaegbulam et al. 2020[7]	Case Series	COVID-19, N=4, 56.3 years	1000 IU cholecalciferol daily (standard dose) or ergocalciferol 50,000 IU daily for 5 days (high dose)	Patients that received a high dose of vitamin D supplementation achieved normalization of vitamin D levels and improved clinical recovery evidenced by shorter lengths of stay, lower oxygen requirements, and a reduction in inflammatory marker status	
Cereda et al. 2020[8]	Prospective observational study	COVID-19, N=324, 38 (11%) treated	Vitamin D supplementation; 58,846 IU/month	hospitalization or mortality	Vitamin D not associated with either hospitalization or inhospital mortality, although a trend toward a 2-fold higher risk of death was associated with vitamin D
Cangiano et al. 2020[9]	Observational study, 2 months	COVID-19, N=157, 63% of subjects tested positive for SARS-CoV-2 on the nasopharyngeal swab test. 37%	Previous vitamin D supplementation	A range of risk factors and clinical characteristics were correlated with outcome	Mortality was inversely associated with vitamin D supplementation

		remained negative			
Ling et al. 2021[10]	A cross-sectional multi-centre observational study, 7 weeks	COVID-19, N=986 (151 treated), aged 74 years	High dose booster therapy: approximately ≥ 280,000 IU in a time period of up to 7 weeks	Mortality	Treatment with cholecalciferol booster therapy reduced mortality OR: 0.38 (0.17–0.84) p=0.018
Giannini et al. 2021[11]	Retrospective analysis, 14 days	COVID-19, N=91 (36 treated), aged 74 years	200,000 IU (×2) cholecalciferol	Transfer to ICU/death	80% reduction in outcome by vitamin D (patients ≥3 comorbid diseases (OR=0.18; 95% CI: 0.04–0.83)
Gönen et al. 2021[12]	Retrospective analysis and prospective study, 14 days	COVID-19, N=867; 163 treated	224-500,000 IU Cholecalciferol/14 days	COVID-19 cases (no comorbidities, no vitamin D treatment, 25(OH)D <30 ng/mL) had 1.9-fold increased risk of having hospitalization greater than 8 days vs. patients with with comorbidities and vitamin D treatment. Vitamin D treatment decreased the mortality rate 2.14-fold.	

*in treated arm. CI = confidence interval; HR = hazard ratio; ICU = intensive care unit; IU = international units; OSCI; Ordinal Scale for Clinical Improvement; OR = odds ratio; RR = relative risk.

References

1. Murai, I. Effect of Vitamin D3 Supplementation vs Placebo on Hospital Length of Stay in Patients with Severe COVID-19: A Multicenter, Double-Blind, Randomized Controlled Trial | MedRxiv Available online: https://www.medrxiv.org/content/10.1101/2020.11.16.20232397v1 (accessed on 21 November 2020).
2. Rastogi, A.; Bhansali, A.; Khare, N.; Suri, V.; Yaddanapudi, N.; Sachdeva, N.; Puri, G.D.; Malhotra, P. Short Term, High-Dose Vitamin D Supplementation for COVID-19 Disease: A Randomised, Placebo-Controlled, Study (SHADE Study). *Postgrad Med J* **2020**, doi:10.1136/postgradmedj-2020-139065.
3. Entrenas Castillo, M.; Entrenas Costa, L.M.; Vaquero Barrios, J.M.; Alcalá Díaz, J.F.; López Miranda, J.; Bouillon, R.; Quesada Gomez, J.M. "Effect of Calcifediol Treatment and Best Available Therapy versus Best Available Therapy on Intensive Care Unit Admission and Mortality among Patients Hospitalized for COVID-19: A Pilot Randomized Clinical Study." *J Steroid Biochem Mol Biol* **2020**, *203*, 105751, doi:10.1016/j.jsbmb.2020.105751.
4. Annweiler, C.; Hanotte, B.; Grandin de l'Eprevier, C.; Sabatier, J.-M.; Lafaie, L.; Célarier, T. Vitamin D and Survival in COVID-19 Patients: A Quasi-Experimental Study. *J Steroid Biochem Mol Biol* **2020**, *204*, 105771, doi:10.1016/j.jsbmb.2020.105771.
5. Annweiler, G.; Corvaisier, M.; Gautier, J.; Dubée, V.; Legrand, E.; Sacco, G.; Annweiler, C. Vitamin D Supplementation Associated to Better Survival in Hospitalized Frail Elderly COVID-19 Patients: The GERIA-COVID Quasi-Experimental Study. *Nutrients* **2020**, *12*, doi:10.3390/nu12113377.
6. Tan, C.W.; Ho, L.P.; Kalimuddin, S.; Cherng, B.P.Z.; Teh, Y.E.; Thien, S.Y.; Wong, H.M.; Tern, P.J.W.; Chandran, M.; Chay, J.W.M.; et al. Cohort Study to Evaluate the Effect of Vitamin D, Magnesium, and Vitamin B12 in Combination on Progression to Severe Outcomes in Older Patients with Coronavirus (COVID-19). *Nutrition* **2020**, *79–80*, 111017, doi:10.1016/j.nut.2020.111017.
7. Ohaegbulam, K.C.; Swalih, M.; Patel, P.; Smith, M.A.; Perrin, R. Vitamin D Supplementation in COVID-19 Patients: A Clinical Case Series. *Am J Ther* **2020**, e485–e490, doi:10.1097/MJT.0000000000001222.
8. Cereda, E.; Bogliolo, L.; Lobascio, F.; Barichella, M.; Zecchinelli, A.L.; Pezzoli, G.; Caccialanza, R. Vitamin D Supplementation and Outcomes in Coronavirus Disease 2019 (COVID-19) Patients from the Outbreak Area of Lombardy, Italy. *Nutrition* **2021**, *82*, 111055, doi:10.1016/j.nut.2020.111055.
9. Cangiano, B.; Fatti, L.M.; Danesi, L.; Gazzano, G.; Croci, M.; Vitale, G.; Gilardini, L.; Bonadonna, S.; Chiodini, I.; Caparello, C.F.; et al. Mortality in an Italian Nursing Home during COVID-19 Pandemic: Correlation with Gender, Age, ADL, Vitamin D Supplementation, and Limitations of the Diagnostic Tests. *Aging (Albany NY)* **2020**, *12*, 24522–24534, doi:10.18632/aging.202307.
10. Ling, S.F.; Broad, E.; Murphy, R.; Pappachan, J.M.; Pardesi-Newton, S.; Kong, M.-F.; Jude, E.B. High-Dose Cholecalciferol Booster Therapy Is Associated with a Reduced Risk of Mortality in Patients with COVID-19: A Cross-Sectional Multi-Centre Observational Study. *Nutrients* **2020**, *12*, doi:10.3390/nu12123799.
11. Giannini, S.; Passeri, G.; Tripepi, G.; Sella, S.; Fusaro, M.; Arcidiacono, G.; Torres, M.O.; Michielin, A.; Prandini, T.; Baffa, V.; et al. Effectiveness of In-Hospital Cholecalciferol Use on Clinical Outcomes in Comorbid COVID-19 Patients: A Hypothesis-Generating Study. *Nutrients* **2021**, *13*, doi:10.3390/nu13010219.
12. Gönen, M.S.; Alaylıoğlu, M.; Durcan, E.; Özdemir, Y.; Şahin, S.; Konukoğlu, D.; Nohut, O.K.; Ürkmez, S.; Küçükece, B.; Balkan, İ.İ.; et al. Rapid and Effective Vitamin D Supplementation May Present Better Clinical Outcomes in COVID-19 (SARS-CoV-2) Patients by Altering Serum INOS1, IL1B, IFNg, Cathelicidin-LL37, and ICAM1. *Nutrients* **2021**, *13*, 4047, doi:10.3390/nu13114047.
